# An exploration of health workers’ experiences in providing bereavement care to mothers following a stillbirth: results from a subnational level health system in Uganda

**DOI:** 10.1186/s12884-023-05913-x

**Published:** 2023-08-17

**Authors:** Eric Ssegujja, Isaac Ddumba, Michelle Andipatin

**Affiliations:** 1 Department of Health Policy Planning and Management, School of Public Health, College of Health Sciences, Makerere University, Kampala, Uganda; 2 School of Public Health, Faculty of Community and Health Sciences, University of the Western Cape, Cape Town, Republic of South Africa; 3 Mukono District Local Government, Mukono, Uganda; 4 Department of Psychology, Faculty of Community and Health Sciences, University of the Western Cape, Cape Town, Republic of South Africa

**Keywords:** Stillbirth, Maternal health, Bereavement care, Health workers

## Abstract

**Background:**

Stillbirth is a profound emotion-laden event to the mothers and health workers who provide care due to its sudden and unexpected occurrence. Health workers offering support in regions shouldering the highest-burden experience providing support to a stillbirth mother in their professional lifetime. However, their experiences seldom get documented as much of the focus is on mothers causing a dissonance between parental and clinical priorities. This study aimed to explore the health worker’s experiences in the provision of bereavement care to mothers following a stillbirth.

**Methods:**

An exploratory cross-sectional qualitative study was undertaken on a purposively selected sample of key informants drawn from frontline health workers and health systems managers providing maternal health services at a subnational level health system in Uganda. An interview guide was used to collect data with the audio-recorded interviews transcribed using Microsoft office word. Atlas. ti a qualitative data management software aided in coding with analysis following a thematic content analysis technique.

**Results:**

There was no specialised bereavement care provided due to inadequate skills, knowledge of content, resources and support supervision for the same. However, health workers improvised within the available resources to comfort mothers upon news of a stillbirth. Disclosure to mothers about the stillbirth loss often took the form of forewarnings, direct and sometimes delayed disclosure. A feeling of unpreparedness to initiate the disclosure process to the mother was common while the whole experience had an emotional effect on the health workers when establishing the cause, particularly for cases without clear risk factors. The emotional breakdown was often a reflexive response from the mothers which equally affected the care providers. Health workers engaged in comforting and rebuilding the mothers to transition through the loss and validate the loss. Efforts to identify the skills and health systems gaps for address were a common response targeted at improving the quality of maternal healthcare services to avert similar occurrences in the future.

**Conclusion:**

Providing care to mothers after stillbirth was an emotional and challenging experience for health workers requiring different approaches to disclosure and provision of emotional support. The aspect of specialised bereavement care was lacking within the current response. Reflection of unpreparedness to handle the tasks demonstrates a deficit in the required skills. It is a critical gap missing hence calling for dedicated efforts to address it. Targeting efforts to improve health workers’ competencies and preparedness to manage grieving mothers is one way to approach it.

**Supplementary Information:**

The online version contains supplementary material available at 10.1186/s12884-023-05913-x.

**Table:** 

**Text box 1.** Contribution to literature
• Providing care to mothers after stillbirth is challenging for health workers but it yet to receive the attention required in literature.
• Consequently, a gap between current health worker’s experience in provision of care after stillbirth and public health interventions which informs practice exists and more pronounced in sub-Saharan Africa, a region with a high burden.
• No specialised bereavement care provided due to lack of skills, knowledge on content, resources and support supervision for the same.
• Unpreparedness, emotional effects and a feel of inadequate skills by the health workers characterised the process.
• Attempts to comfort and rebuild the mother to transition through the loss, identifying skills and health systems gaps for redress formed the immediate responses to boost confidence in service provision.
• Health workers competencies were challenged during the provision of care to mothers after stillbirth, highlighting the need for tailored skilling to equip them to improve the quality of care.

## Background

A stillbirth is a profound event for parents but also a challenging moment for health workers [1]. It triggers both grief and emotional turmoil and much of the literature speaks to the lived experiences of the mother. To date, there is a dearth of evidence about its effects on health workers who provide care, especially in sub–Saharan Africa which shoulders the biggest burden. The provision of support to mothers after stillbirth is often an emotion-laden experience for health workers [2, 3]. The sudden and unexpected occurrence makes it a very traumatic event. Its effects on health workers providing care may include anxiety, emotional distress, and mental breakdown [3]. It may also create doubt in the efficacy of applied interventions, confidence in health workers’ competencies, the accuracy of information responding to risk factors as well as a disrupted provider-patient relationship [4, 5]. Besides, caring for grieving mothers can instil fear, especially where blame is apportioned to the very health worker who provided the initial care. The anxiety and emotional involvement may also arise due to perceptions that at some moment in their lives, health workers could themselves or their loved ones experience the same [6].

Health workers play a crucial role in the delivery of quality maternal healthcare services many of whom experience the provision of care to a stillbirth mother in their lifetime [7]. Those in regions with a high burden, may over the years gain practical experience in providing care to grieving mothers [8]. However, documentation of such experiences has been suboptimal presenting challenges to the adoption of evidence-based practice. In a bid to improve women’s experience during care and the quality of maternal health, World Health Organisation (WHO) has developed guidelines for the provision of quality maternal health services including those responding to the mother’s emotional well-being [9–11]. These include; Reproductive Maternal Newborn and Child Health (RMNCH) Quality of care guidelines, Patient-centered care guidelines, and respectful maternal and child health services to mention but a few. In the same way, guidelines for the improvement of human resources for health and well-being do exist [12, 13]. This is in recognition that health workers who spend more time caring for mothers may not take adequate efforts to respond to their emotional well-being arising from the emotional impact of the services they provide. However, an international consensus on stillbirth bereavement care is yet to be reached [14]. Bereavement care denotes support offered to mothers following a stillbirth. Although efforts to address this have been ongoing for some time, [15] recent developments appear to have zeroed down on eight principles for stillbirth bereavement care [14]. These include; stigma reduction, respectful care, support for grief response, provision of physical and psychological needs as well as informed choices for future reproductive health among others. Even then, implementation context continues to be the main barrier to mass rollout and adoption, especially in limited resource settings where inadequate resources among others still impede their effective implementation. Indeed, the absence of guidance partly informed the UNICEF/WHO statement on respectful bereavement care following a stillbirth [16].

Interventions to support health workers include welfare schemes to address work-related burnout, psycho-social support as well as health and emotional wellbeing [17, 18]. Implementation has been in varying contexts in anticipation of improved health worker productivity [19]. However, despite the existence of such interventions, little is known about similar efforts to equip health workers for improved service provision to mothers after stillbirth. Studies investigating this have been predominantly in developed country contexts [20, 21]. Nonetheless, these studies provide useful insights reflecting health workers’ expression of unpreparedness to provide support to grieving mothers after stillbirth [4]. A lack of adequate knowledge, mentored experience, communication skills, and personal support affected the confidence with which they offered care [22]. As a result, some were reported to consider quitting the profession while others experienced long recovery processes [10].

Within the Ugandan context, literature has documented different factors influencing health workers’ delivery of care to mothers. Overall, Mills et al. report that health systems’ response to parents after stillbirth remains inadequate [23]. Whereas health workers continued to provide care, sometimes it did not leave a positive effect on parents’ experience of care with major gaps in communication, inadequate resources and workplace pressure on health workers affecting care to mothers [23, 24]. Staff shortages magnified the health worker’s dilemma of who to prioritise in case of the presence of other mothers with live babies and one with a stillborn [25]. Scarcity of supplies, health worker motivation and inadequate infrastructure to permit privacy rather than isolation for mothers were the other factors [26]. Challenges extended into the poor and casual communication of death, uneasiness with disclosure and the use of family members to break the news of a stillborn [27]. Relatedly, some women learnt about stillbirth by overhearing health workers whisper amongst themselves. In related instances, health workers spent a minimal amount of time after disclosure which shortened the opportunity to console and allow the mother to be listened to, spend time with the baby and make memories of the same [27]. With minimal documentation of local evidence and a lack of a structured support system, health workers’ coping strategies are likely to vary by context [22]. Whether current practices meet health worker needs to provide adequate care to mothers experiencing a stillbirth, remains unclear [10]. Learning from their experiences about support to mothers could provide useful insights. This paper aimed to explore the health workers’ experiences in the provision of bereavement care to mothers following a stillbirth at a subnational health system level in Uganda.

## Methods

### Study design

Details regarding the study methodology have been published elsewhere [28] but briefly, the study adopted an exploratory cross-sectional qualitative design. This manuscript explores the health workers’ experiences with the provision of bereavement care to mothers that experienced a stillbirth.

### Study setting

The study was conducted between January to March 2019 at a subnational level health system. Mukono district was selected as a case for an in-depth study of the phenomenon because it represented districts with a relatively high burden of stillbirth at the time. In terms of health service delivery, the district is covered with a total of one hospital, three HCIVs, fifteen HCIIIs, and thirty-two HCIIs. There is a strong presence of the private sector involvement in health service provision with both Private-for-Profit (PFP) and Private-not-for-Profit (PNFP). Maternal health services are offered at all levels of clinical service provision from HCII (outpatient maternal health care) up to the Hospital level with referrals recommended outside the study area for complicated cases requiring tertiary maternal health care at regional and national referral hospitals. Stewardship of maternal health service delivery is directly under the Assistant District Health Officer (A-DHO) in charge of MCH a position designated for a senior nurse/midwife under the Ministry of Health and district local government guidelines.

### Study population

The study population comprised the health workers involved in the delivery of maternal health services within the subnational health systems. Given the pluralistic nature of the health system at this level, the study purposed to have a blend of representation from both the public sector and the private represented by the private-not-for-profit maternal health service providers. These included; frontline health workers, health facility managers, subdistrict health managers, and the District Health Management Team (DHMT).

### Sample and sampling procedure

The study purposively interviewed sixteen respondents drawn from medical officers (n = 5), nurses (n = 5), and midwives (n = 6). Respondents were drawn from a purposive sample of six health facilities and the district health management team. The health facilities included two hospitals, two Health centre IVs and two Health centre IIIs. According to sample distribution, they were drawn from; the district health management team (n = 3), hospital-level (n = 6), Health centre IV (n = 5), and Health centre III levels (n = 2). According to ownership, the two hospitals were private-not-for-profit facilities affiliated with the Catholic Medical Bureau (CMB) and the Uganda Protestant Medical Bureau (UPMB) respectively while the remaining health facilities were all publicly owned. Priority was given to those health workers who were directly involved in the delivery of maternal health services. Details of respondent’s categories are provided in Table 1 below.

**Table 1:** Respondent’s characteristics

Cadre	Level of service provision				Totals
	DHMT	Hospital	HCIV	HCIII	
Medical officers	1	2	2		5
Nurses	2	2	1		5
Midwives		2	2	2	6
**Totals**	**3**	**6**	**5**	**2**	**16**

*Abbreviations: DHMT; District Health Management Team, HCIV; Health Centre Four, HCIII; Health Centre Three.*

### Data collection process

Data collection was conducted by the first author (ES) an experienced qualitative researcher with working knowledge and experience of the study area’s health systems functionality. He was assisted by a female graduate-level research assistant also conversant with qualitative methodologies and familiar with the local health systems especially as it related to the delivery of maternal and child health service delivery. After introducing the study to the district health office, consultations were made about potential respondents. This led to a compilation of a list of potential respondents with their contacts to be approached later by the study team. The same process was repeated at each of the health facilities that were visited. Potential respondents were then approached with the purpose of the study explained. For those that expressed willingness to participate, a date, time and secure venue for the interview were agreed upon. On the day of the interview, face-to-face interviewer-administered interviews with managers and frontline health workers directly involved in clinical and managerial decision-making were conducted at their respective places of work. Overall, none of the contacted potential respondents declined to participate. Despite putting in place measures to manage the potential for distress during the interviews, we did not experience any incidence that required such an intervention. Broadly the interview guide was developed for this study and informed by the literature. All interviews were conducted in English and lasted between 45 min to one hour and were audio-recorded with digital recorders with notes taken during the interviews. After the sixteenth interview, no new data insights were emerging and the study team assumed to have attained data saturation where no additional interviews we conducted beyond that point. At the end of each field day, field notes were expanded and recorded data were downloaded onto a password-protected computer with a copy saved on the external hard drive.

### Data analysis

This manuscript utilised secondary data from a larger study and followed a thematic content analysis technique. All audio interviews were transcribed verbatim into Microsoft office word by the first author and a graduate-level research assistant who had participated in data collection. We did not return the transcripts to respondents for comments. Thereafter they were entered into Atlas. ti a qualitative data management software [29]. A codebook was developed by the study team following an inductive coding of five sample transcripts to identify emerging themes and subthemes. These were discussed with the study team to come up with the codebook that guided an inductive coding process on all the transcripts. After a thorough reading through each of the transcripts, textual data relating to each of the themes in the codebook was highlighted, and assigned to each of the codes. Query reports were run for each of the codes and a manual pile sorting process involved grouping text with similar meaning into the same piles by the first author (ES). These were further analysed inductively for emerging sub-themes. Triangulation involved a comparison of results from the audio transcripts with field notes as well as a contrasting of textual data from the different categories of respondents. Feedback on the contextual interpretation of results was provided by the second author (ID) while the input to the codebook development, coding process, theme development and triangulation of results was provided by the third author (MA). Summarised mini-statement were then used to represent each of the subthemes. These have been used in the presentation of results as reflected in the results section to bring out the respondents’ voices. Finally, we followed the Consolidated Criteria for Reporting Qualitative Research (COREQ) while reporting the qualitative results (attached here as Additional file 1).

### Researcher reflexivity

ES is a health systems researcher who before this study worked as a project coordinator on a five-year district management capacity strengthening intervention that aimed at reducing the burden of childhood illnesses in several districts including the study district. While working on the said project, he interacted with health systems managers regularly where he came face-to-face with implementation challenges inherent in the study area as they regarded the delivery of maternal and child health services. ID works with the subnational health systems where he leads the implementation of maternal and child health interventions and is actively engaged in health systems research but did not participate in data collection. This may have influenced their perspectives on themes during study implementation and data analysis. During data collection, the team introduced themselves as researchers with the objectives of the study clearly stated upfront as intended for academic purposes.

### Patient and public involvement

Patients and members of the public were not involved in the study design, setting of the research questions, data collection and analysis, interpretation or writing up of results, or reporting of the research.

## Results

Overall, packaging of disclosure information following a stillbirth focused on the immediate interventions available to support the mother. A general feeling of unpreparedness was common across respondents. Health workers too sometimes became vulnerable to emotional breakdown while managing the situation. Challenges were reported while attempting to establish the likely causes of delays to reach the facility. Emotional support to the mothers were a common reflect among respondents including provision of support which reflected empathy. A delicate relationship existed with the mothers while rebuilding them to transition through the loss with strategies adopted including identification and navigating avenues to address perceived gaps which may have contributed to the loss.

Inadequate knowledge and skills to provide postpartum care to stillbirth mothers, loose relationship post discharge which minimises any possible efforts for a follow-up to address the care concerns, challenges in providing feedback on what could have led to the stillbirth, postpartum counselling on how to negotiate subsequent pregnancies and inadequate contact between the health worker and mother postpartum were among the numerous challenges as reflected in the quotation below;

*“because this woman who gets a stillbirth goes home with no knowledge of what to do next. She will not come for post-natal care because she has no child to immunize, she doesn’t know what happened to this one, whether she should get another baby and when, nobody sits her down to tell her”.****Nurse Manager***.

Overall, no specialised bereavement care was provided due to a lack of skills, knowledge on content, and absence of guidelines, resources and guidance on support supervision for the same. Common across all experiences was the absence of standards in the bereavement support that health workers offered to the mothers. Rather, segmented efforts of individual health workers that were attending to the bereaved mothers at the time reflected bereavement support. Health workers improvised within the available resources to comfort mothers upon news of a stillbirth. While at the initial stage of diagnosing a stillbirth, health workers endeavoured to come to terms with the outcome before engaging the mother. Eventual disclosure took different forms including forewarning as cues to what was yet to happen, direct and sometimes delayed. A feeling of unpreparedness for the next engagement was reported. Provision of emotional support was observed in the health worker’s immediate response but would occasionally take a negative effect on the health worker’s emotional well-being too. These emerged as immediate reflexes before health workers engaged in comforting and rebuilding the mothers to transition through the loss while paying attention to their special needs. Results also show that health workers embarked on identifying the skills and health systems gaps that could have contributed to the outcome. This was targeted at addressing the root cause to improve the quality of maternal health care to avert similar occurrences in future. Later we elaborate on each of these results in detail but first is the summary presentation of themes and subthemes in Table 2 below.

### Experiences with disclosure

Packaging of the disclosure message following a stillbirth diagnosis was tailored to the immediate interventions focusing on the mother. The processes involved linking the messages to the causes familiar to the mother and the appropriate time when to disclose the outcome would be made. Results revealed that disclosing to the mother took different dimensions including forewarnings to the mother about the presence of potential risks before the actual stillbirth event. The use of herbal concoctions to induce labour and relieve pain was specifically mentioned as a common practice that often resulted in abnormal contractions. Once identified, health workers took efforts to caution parturient mothers about the potential risk including stillbirth.

*for those mothers who get fresh stillbirths in most cases, you would have talked to them earlier. Sometimes the mother is uncooperative while pushing or in other instances, you would have caught her already taking some herbal medication, and I warn them that you might lose your baby because of these herbs I have found you taking.* ***Health manager at HCIII***

The main task revolved around letting the mother know about the outcome of the pregnancy. Following a diagnosis of a stillbirth, a common theme identified while disclosing to the mother centred on the cause. Under such circumstances, health workers were observed to take on a “tell it all” approach when the perceived cause rested less on the health worker. The information delivered would be based on the cause;

*I base it on the cause of the stillbirth and if it was the baby being distressed. Others come here when they have failed from somewhere else and come when they even have medical notes and cannulas. The mother would narrate that I was put on a drip.* ***Health manager at HCIV***

### A feeling of unpreparedness to break the news

A feeling of being caught off guard and unprepared for the outcome and to deliver the news ran through most of the responses. In some instances, health workers would break the news to the mother themselves or call in a colleague to support the disclosure process. In one of the interviews, a respondent narrated the process of passing over the disclosure midway through the process to other health workers to complete;

*After doing that you have to counsel this mother or you call the doctor or you go and see the doctor and explain to him that this mother has this and that and I have done this and that. So, you need to take it on from there so that when the doctor takes over he has to do his part by managing this mother.* ***Midwife***

Instances of delayed disclosure by the health workers were reported. This was especially the case where health workers concluded that the mother was not yet ready to receive the message as it was reported during one of the interviews by the maternity in-charge;

*what do we do if she is from theatre and we have tried and failed, from the theatre we will tell her the truth especially mothers under spinal anaesthesia you tell her you have lost the baby so she will cry. If the anaesthetist tells you that don’t tell her we will not do it until she stabilizes.* ***Maternity in-charge***

The process did not go without challenges, respondents revealed instances when the initial disclosure that had gone successfully would later be met by an emotional breakdown when the grieving mother set sight on her peers holding babies. It took an emotional burden on some of the health workers who had to once again repeat the preparation of the mother emotionally, especially those that took longer in denial.

*when she is still in the delivery room before seeing her friends, she is ok but when she sees her friends with babies that is where the problem starts. Sometimes you leave the delivery room when you have agreed on everything and, in most cases, if it’s IUFD you tell them immediately but some say that my baby is still alive and I have been feeling the fetal movements and yet it’s a macerated stillbirth.* ***Nurse***

### The emotional effect on the health worker

Health workers experienced difficulty while making meaning of the loss within themselves in closely related ways the mothers felt. Connecting with the mother while delivering news of the stillbirth loss for which they were equally not sure about the cause made the provision of immediate explanation particularly challenging.

*Somebody comes and throughout antenatal the baby is ok and the expected date passes the mother comes back with an IUFD and you ask what has caused this and the mother can’t explain and she is like you told me to come back when contractions start and I have come back when they have started and the baby is dead.* ***Midwife***

It was a particularly shocking experience especially when health workers had similar personal stories they could relate with. Some health workers, they had interacted with close friends who had experienced a stillbirth in the recent past. When the same happened at their health facilities, it left them wondering what the cause could have been. Narrating this experience, a respondent thus noted;

*I realized it from my personal friends, and then coming here I also realized that women experienced it from our health facility so I was wondering what is causing it. But since it only happened last month we were still investing in what could have been the cause of that.* ***Health manager***

Even worse was the experience when the health workers providing care were pregnant at the time. In one particular instance, a pregnant midwife found it difficult while they provided care to mothers who had experienced stillbirths.

*I received a very bad experience because I received four mothers on the same day, they all lost their pregnancies and we were all of the same gestation age (26 weeks). And their babies all died, we had a fresh stillbirth, and the same day we got a macerated stillbirth so it was bad.* ***Hospital midwife***

### Establishing the Cause of Stillbirth

Health workers experienced challenges while establishing the likely causes of delay to reach the health facility by the mothers. To provide adequate counselling, required mothers to give detailed information about their care seeking before admission. However, it emerged that some would conceal information regarding where they first sought care from. This impeded efforts by health workers to guide mothers well on appropriate care seeking. In some cases, such information only emerged after the discharge of the mother which made it impossible to offer appropriate guidance. Health workers indicated that reasons for possible delays would emerge from other admitted mothers.

*sometimes these mothers don’t tell you the truth of where they started from …. But after delivery, the other mothers with whom she was on the ward will be the ones to tell you that the mother who has just gone said that they started from the Traditional Birth Attendants (TBA).* ***Health manager at HCIV***

Consultations among health workers to establish the cause, especially among the ones who were present upon admission were reportedly undertaken. Such processes would sometimes be conducted outside the formal perinatal death review proceedings. In a bid to seek clarity about the delayed referrals, health workers extended the inquiry to establish the lower-level facilities the mother attended last before the eventual referral. In one instance, facility in-charges would initiate contacts with such private facilities to establish the triggers.

*some of the facilities around us, we know and the staff working there we know so if they see that a mother had obstructed labour they call the health facility and we use our airtime to find out what happened. Sometimes the midwife can tell you that the mother was referred at such and such a time and we can compare the time of referral and time of arrival at the facility. If I fail on the phone sometimes, we meet and discuss, [and inform them] you sent us a mother with such and such a condition and the outcome was this.* ***Maternity In-charge***

### Intervening to support the mother

#### Ensuring emotional presence for the mother

Emotional support to the mother was a common reflex to news of stillbirth was reported. It formed part of the care provided to women. After disclosure, health workers ensured the emotional presence of the mother. This involved emotionally relating with the mother to support her to overcome emotions and grief. The care involved sharing mutual emotions of loss to the mother, health workers and the health facility in general. Other strategies involved remaining available to the mother after discharge which was reflected in the provision of maternal health advice beyond the postnatal period and continued support during the subsequent pregnancy.

*After counselling her and we see that she has accepted it and we start working [with] her telling her that it is not the end of her life and we are trying to save her life now.* ***Midwife***

Despite efforts to deliver quality services, there were instances when mothers expressed anger, and disappointment and sometimes blame the health workers for having contributed to the outcome. Respondents expressed a lack of adequate skills to provide the much-needed emotional support to the mothers in cases where the blame was directed at the health workers as revealed in the quote below;

*there is a gap when it comes to counselling these mothers. So, when it comes to the counselling of these mothers it is difficult in two ways on the side of the health workers and even the mother. You know after such an experience it is very difficult to sit down with this mother and in most cases, she might even be blaming you for the death of her child, and yet you are the same person who is counselling her and then telling her to come back is not easy because she might have been attending antenatal from your facility.* ***District Health Manager***

#### Understanding the loss and attached meaning to the mother

Health workers provided support to mothers which reflected empathy. It involved counselling mothers who expressed reflexive anger due to the inability of the health facility to save their babies. Such experiences often invoked feelings of self-doubt arising from the health workers’ acknowledgement of the health facility’s potential to save the unborn baby. Meaningful emotional support included the provision of assurance that once risk factors were averted, there would be a possibility for mothers to receive good care for subsequent pregnancies.

*we normally do counsel any other who has lost a baby and we stress important areas that you should observe during their next pregnancy as a way of trying to curb a re-occurrence of this event*. ***Medical officer***

Setting sight on other women holding babies would elicit rage and anger among some mothers who had a stillbirth. In some instances, they would express the desire to vacate the facility immediately. This meant health workers would adjust the counselling session to suit the minimum available time given by the mother. The rush to provide emotional support compromised the quality of care as was reflected in one of the interviews;

*yes and since we have a high number of women when one sees that hers didn’t survive and the others are having their babies, she becomes desperate and by the time you think of sitting and completing this form, she will just tell you that musawo [health worker] I will come back to complete this form they want to take the dead body and we are going very far.* ***Health manager at HCIII***

The impact of the loss extended beyond the health worker to reflect negatively on the health facility’s performance in general. Respondents observed that stillbirth occurrences cast the health facility in a bad light as seen in the quotation below;

*it is a hurting moment for the hospital and actually, as trained medical personnel we would wish to see mothers go back with their babies so it’s a bad experience for the hospital. Because as you can see we have big numbers like in 6 months we have got 30 cases. It is not good for us and as medical personnel, it is not good to see a mother who has carried the pregnancy for nine months go without a baby.* ***Hospital Midwife***

### Comfort and rebuilding the mother

A delicate relationship after a stillbirth event surrounded the interventions health workers introduced to support the mother. Anxiety about succeeding with the proposed intervention was among the stressors they had to deal with while providing meaningful care. It emerged that instances of identifying a private and quiet space from where to counsel the mother were a common practice in settings with such resources available onsite. Since this was not a usual counselling session, respondents indicated paying attention and providing extra care while conducting the counselling sessions.

*when counselling this mother, you have to handle her very carefully because she has lost a child and she can change there and then and at last she will end up crying so you have to counsel her*. ***Midwife at HCIV***

#### Protective of the mother’s needs

In navigating ways to support the mother, health workers took care of their medical and psycho-social needs. A case was reported where mothers who delivered through caesarean section were retained at the health facility and requested their caretakers to stay until full recovery both emotionally and medically. The opportunity provided health workers with ample time to provide adequate care to the mothers.

*if it was a c-section they will retain the mother around for the healing to take place. Then we usually try to put them in an isolation side which doesn’t have mothers with babies because sometimes they go into a bit of depression here and there when they hear the babies cry.* ***Medical officer***

Paying attention to cultural sensitivity surrounding mourning of such a loss was also reported. It emerged that immediate burial practices without adequate mourning would not permit mothers to stay for long at the health facility. Some preferred not to reveal too much information thereafter. Attempts to collect as much information and provide counselling would sometimes end in futile efforts. Instances of family members hurrying the health workers through any possible intervention characterised by less attention from the mother were reported;

*these women who lose their babies sometimes don’t want to reveal much of their information. They are in most cases desperate after knowing that the baby has died. So when you divert them to complete the forms they will be rushing you [through] that we want to take our baby since it didn’t cry the people supposed to bury are old and they want to go.* ***Midwife***

#### Supporting mothers to transition through the loss

Health workers reported facilitating a process for the mothers’ transition from the loss to a meaningful life after. The common thread cutting across transitional empowerment was discussions around subsequent conception together with the partner. For mothers who were initially in denial, the first step involved supporting them to accept and negotiate around the loss. It emerged that while initiating guidance on how to maintain the next pregnancy healthy, health workers would also discuss the need for the mother to make adequate spacing for the next conception;

*I usually tell them to at least take some amount of time before conception and if they do get pregnant again things may actually be worse than this and they may not be lucky again. We always tell them to take at least 2–3 years before they conceive again.* ***Medical officer***

Transitioning out of the loss extended to the health workers who provided care. Respondents reported receiving mentorship from peers to gain skills in responding to identified gaps and addressing similar cases in future. A step-by-step process of reviewing the documentation about a particular case was followed to identify skills gaps and address them.

*and if you get a Fresh Stillbirth (FSB) we read through it and get to know what happened. If we get a Macerated Stillbirth (MSB) the midwife has to know that she has to check for the BS and check for other conditions. So, every day we learn so I follow them up if you had an MSB on your duty I don’t want you to get an MSB on another duty. If it happens then you have a problem, not you but there is a problem. So that is how I do my things.* ***Maternity in-charge***

### Addressing perceived gaps

Various strategies were adopted while navigating ways to respond to identified gaps. The focus was reflected on both the mother and the health workers who provided care. Such occasions were used as platforms to pass on technical information to respond to similar cases in the future;

*we check ourselves and if it was the health worker who was reluctant, next time we have to change. And if it was on the mother’s side, we give health education talks during antenatal such that these mothers attend antenatal early.* ***Medical officer***

For internal capacity building within the facility, the provision of feedback to health workers was another avenue of supporting them with the required skills. This was reported to happen during staff meetings guided by the review outcomes. The provision of feedback was another way of facilitating health workers’ growth. It also emerged that often health workers would make efforts to find out what the findings of the inquiry were. A respondent thus noted;

*whenever we finish the reports we give the feedback to our staff. Every Wednesday we have a meeting so if we finish the report on Monday, we have to give them a report on Wednesday. …Someone can ask what happened then? you tell them that we got a referral for the ruptured uterus. So, all in all, you can understand that it wasn’t us but it was external.* ***Health manager***

Despite the challenges, health workers maintained confidence in their ability to address the gaps. They observed that at the time, no amount of preparation would empower them to handle the situation in varying contexts. Fear of having to shoulder the blame was one of the underlying factors. In one instance, a respondent noted that being blamed by the mothers and loss of confidence in the services provided were common.

*even if you get one stillbirth, no one would like to lose their baby. When you get a stillbirth as a health worker the mother in most cases will cast the blame on you. So even our health workers don’t want to get stillbirth be it macerated or fresh. There is that loss and the mother will lose confidence in our services/facility as the people that handled her. So, we also try very much to ensure that we go deep to know what the cause was.* ***Maternity In-charge at HCIII***

## Discussion

Our results suggest that health workers go through challenging experiences while providing bereavement care to grieving mothers after experiencing a stillbirth at a subnational level health system in Uganda. Overall, there was no standard procedure for the provision of bereavement care provided to the mothers. The absence of the required skills, resources, care content and support supervision impeded on the level of preparedness for the health workers to offer the same. Rather, health workers improvised through the available resources to support and conform mothers through their loss. The research broadly embraced the improvised care extended to mothers as a process that involved different stages. The research adds to the understanding that caring for mothers after stillbirth within a limited resource setting was an emotional process that put to test the limited skills and capabilities of the handling health worker. Adequately, the research shows that health workers made efforts amidst numerous challenges to provide bereavement care to mothers experiencing stillbirth.

### Disclosure upon stillbirth diagnosis

Tailoring messages that focused on the mother started with the discussion of causes which were familiar to the mother. This involved forewarning about engagement in harmful pregnancy care practices such as the use of herbal concoctions before eventual disclosure of stillbirth outcome was made. The other common strategy reflected a “tell it all” when the cause was well known to the health worker. These strategies may have been adopted as quickly and easily applied strategies of disclosure by the health workers to fend off any potential possibility of blame and accusations directed towards the health worker as having contributed to the cause. It could also have been a way of signalling an alarm or red flag to the mother in a long process of disclosure as a form of alerting her to the reality that the potential for a stillbirth could not be ruled out at that time hence a process to prepare the mother for the unfortunate news “just in case”. The value of health worker interaction with and communication with the mother both in what is said and how it is delivered has been reported elsewhere [23, 25]. They have the potential to minimize parents’ trauma and vulnerability during this period. Poor communication in particular and unsupportive facility practices meant that bereavement care needs were often unmet [23]. Demonstrating sensitivity and empathy while validating parents’ emotions was key to ensuring respect, dignity and culturally appropriate care [22]. As it could be observed, these may not have been the best strategies to communicate with mothers about their stillbirth loss. Other than supporting the mother to cope, the immediate response was reflected in the form of protecting the health worker first then the mother’s emotional well-being and interest later. This could be seen as not situating the mother at the core of bereavement care following a stillbirth. There was an urgent need to equip health workers with adequate skills and resources to provide quality and professional, respectful bereavement care to mothers following a stillbirth [15]. Improved communication during this time is hence important for mothers to adjust and cope with the loss [16].

### The emotional effect on the health worker

Disclosure took an emotional toll on health workers and particularly perturbing where the cause was not obvious and remained unclear, especially in cases where mothers adhered to ANC visits. The experience was even complicated when the handling health worker had previous experience with the same and worse when they were pregnant. The potential for health workers to get emotionally affected by providing care to stillbirth-bereaving mothers could in the immediate term have affected the quality of services they provided. In the longer term, it could have affected their continued provision of service in this area of maternal health hence contributing to the gap. Challenges supporting mothers by healthcare workers have been echoed previously. These include; difficulty dealing with grieving parents, lack of a happy outcome from any form of support offered, mothers’ anger and limited resources [13, 16]. The situation is worsened by a lack of mentoring experience, communication skills, knowledge, and thus confidence deficit and competence in providing sensitive care [13, 16]. Given that the health worker providing this care came from the same context, it was inevitable that they would likely have experienced the same or had a close family/friend go through the same. Therefore, interface with a stillbirth incident was likely to evoke similar emotional reactions in the health worker as they did to the affected mother [30]. Not paying attention to this aspect of care could exacerbate the health and well-being of mothers with the potential for a long-term effect on the mental health and well-being of health workers. At the health systems level, it could ruin the quality of other related maternal and child health services resulting in poor uptake of the same. Targeting capacity building to ensure the strengthening of the emotional well-being and confidence of the health workers to deliver such services could go a long way toward improved quality services specifically targeting bereavement care. Within the study context, it was understandable since some health workers lacked counselling skills, and those who may have learned bits of it, it could have been during their pre-service training. The more readily available counselling training was HIV counselling. The inability to “configure” aspects of this counselling training into maternal and child health services calls for reflection. It is even appropriate that PMTCT as an MCH program laden with counselling aspects is anchored within the HIV services. We recommend that other than developing a wholly new intervention, taking stock of these available skills and leveraging them to address bereavement care after stillbirth would be of greater value.

**Table 2:** Summary of key findings by themes and subthemes

	Key Themes	Subthemes
1	Experiences with disclosure	Unprepared to break the news
		Emotional effect on health worker
		Establishing the cause
2	Support the mother’s recovery	Ensuring emotional presence
		Understanding of loss to the mother
3	Comfort and rebuilding the mother	Protective of mother’s needs
		Support to transition through loss
4	Responding to perceived gaps	Focus on mother knowledge
		Address health worker skills

### Intervening to support the mother

While providing care, health workers ensured emotional presence to the bereaved mothers while sharing mutual emotions of loss with the mothers although it was met with skills inadequacy to provide quality bereavement care. Despite health workers making all good intentions and efforts to support the mother, sometimes they did not go as planned. Elsewhere, similar reports of feeling ill-equipped to support grieving mothers with bereavement care have been reported with well-meaning attempts leading to unintended effects [31]. Similarly, this was attributed to the absence of institutional guidance on how to manage such cases [32]. And for mothers, no amount of preparation could have equipped them with the confidence to manage when they discovered that their baby was stillborn even in regions with high prevalence [23]. Instances of women’s recall of insensitivity and lack of clear and understandable information on the part of health workers while providing care continue to be reported [23]. In other cases, this manifested in the form of delayed or withholding verbal communication of stillbirth, abrupt and intermittent communication, uneasiness with disclosing, and little or no time spent with mothers after disclosure among others [27]. There are ongoing efforts to ensure mothers receive the best bereavement care after a stillbirth. The domesticated WHO quality of care protocols is key in these efforts. However, the delivery of quality services is still impeded by inadequate skills among healthcare workers. Documenting current practices undertaken by health workers to support mothers with bereavement care in different contexts has the potential to inform the kind of interventions appropriate to address this gap.

### Comfort and rebuilding the mother

Efforts to rebuild the mother recover from the loss included paying attention to both their medical and psycho-social needs, cultural sensitivity as well as transition and care after discharge. Within the service provision context, health workers are presented as the only immediate point of care to turn to for support [31]. Consequently, the burden to provide all forms of care rested on the available health workers including support they may not have had the skills to offer to the mothers. Mothers too recognised that the needed support may not have been available and in some instances were surprised that a “stranger” like a health worker could provide quality care and speak to them in a sensitive way [27, 33, 34] with some expressing “relief” at health workers’ actions. The desire for pregnancy increases with the loss of a child and this is especially true when a stillbirth has altered many of the mothers’ plans such as expectations of parenthood and dreams towards a growing family [32]. Irrespective of the quality, the services offered remained the only available intervention for mothers and inevitably would tend to be directed towards the mother’s aspirations and life after the loss. Key aspects of responding to the mothers’ needs must be incorporated into such kind of bereavement care if it is to deliver meaningful recovery to the mother. Since addressing stillbirth has already received global and national recognition as a priority public health challenge, improvement bereavement care to rebuild the mother would also contribute towards this goal.

### Strength and limitations

This study had some strength; first, a purposive sampling strategy was adopted whereby the selected respondents had experienced first-hand providing care for mothers who had experienced a stillbirth. Therefore, the views and opinions for which the results are presented provide a first-hand account of respondent’s experiences. Second, the respondents were drawn from a blend of public and private providers which offered opportunity to learn from both settings. None-the-less, it was not without limitations, first, the social desirability bias was one limitation associated with respondent-provided information. Stillbirth is a traumatizing event not only for mothers but also for health workers. Where the experience was negative and partly attributable to the health worker, bits of information and experiences shared may have skipped out aspects that were not socially desirable. Second, a fundamental shortcoming was not including the voices of mothers that received these services. Maybe their experiences would have helped in triangulating the information provided by the health workers. Had the study included mothers, perhaps we would have reported on their perspectives. Therefore, what is reported here is from the perspectives of the service providers and it should be interpreted in that context. Also, this manuscript is a culmination of additional secondary analysis which was conducted on an already existing dataset. Had we collected these insights during data collection, perhaps there would have been more probing to elicit more information. Lastly, context is an important aspect of health services research. It should be recalled that this study was conducted in a resource-limited setting where some of the factors influencing care provision have a great bearing on resource availability. It is our conviction that the experiences would have played out differently had the health workers been exposed to ample resources to support these mothers. Therefore, caution should be exercised while interpreting these results. Nonetheless, all efforts were made to ensure that these limitations were minimized in such a way that they did not affect the integrity of the results in a substantial manner.

## Conclusion

Our findings show that providing bereavement care to mothers after stillbirth is an emotional and challenging experience for health workers. Given the sensitivity of maternal health, such a loss may instead attract blame and backlash to health workers which could leave them demoralized and demotivated. On top of working to equip them with adequate skills to provide care to bereaved mothers, it’s ideal that compassion is expressed while providing feedback to health workers on how to improve care. Also, it is key that once causes are identified, caution is exercised while providing this feedback and in the long term when collaboratively working together to address the identified gaps.

### Policy implication

integration of bereavement training in medical curricula, setting up guidelines for handling such mothers, institutional support for health worker welfare following perinatal bereavement handling. It is also important to note that the absence of guidelines and documented experiences limits the application of evidence-based practice and consistent delivery of optimal care to women after stillbirth.

### Implications to practice

Improving soft skills such as counselling, and communication skills of health workers, and meeting their psychological needs after providing support to grieving mothers is important while addressing the stillbirth burden in Uganda.

### Recommendation for future research

Research into what happens to health workers who provide care to stillbirth-bereaving mothers is recommended. Such a study could look into; the effects of grief associated with supporting stillbirth-bereaving parents later due to this emotional pain, interventions available to support those health workers, as well as the coping mechanisms with stillbirth loss among health workers among others.

### Electronic supplementary material

Below is the link to the electronic supplementary material.

Supplementary Material 1

## Data Availability

The datasets used and/or analyzed during the current study are available from the corresponding author upon reasonable request.

## Abbreviations

ANC: Antenatal Care
CAO: Chief Administrative Officer
CMB: Catholic Medical Bureau
DHO: District Health Office
DHMT: District Health Management Team
HC: Health Centre
IUFD: Intra Uterine Foetal Death
MCH: Maternal and Child Health
MoH: Ministry of Health
PHC: Primary Health Care
PMTCT: Prevention of Mothers to Child Transmission
PNFP: Private Not For Profit
PFP: Private for Profit
PNFP: Private-not-for-Profit
PPPPH: Public-Private Partnership Policy for Health
PHC: Primary Health Care
QOC: Quality of Care
RMNCH: Reproductive Maternal New-born and Child Health
SB: Stillbirth
TBA: Traditional Birth Attendants
UNICEF: United Nations Children Fund
UPMB: Uganda Protestant Medical Bureau
WHO: World Health Organisation
